# Exploring the Interaction Between Sleep Patterns, Cardiac Autonomic Function, and Traditional Cardiovascular Risk Factors Following Acute Myocardial Infarction

**DOI:** 10.1002/clc.70183

**Published:** 2025-07-24

**Authors:** Mohamed Ali Hbaieb, Laurent Bosquet, Omar Hammouda, Raghda Hbaieb, Ines Mezghani, Salma Charfeddine, Leila Abid, Mouna Turki, Tarak Driss, Benoit Dugué

**Affiliations:** ^1^ Laboratory Mobilité, Vieillissement, Exercise (MOVE) (UR20296), Faculty of Sport Sciences University of Poitiers Poitiers France; ^2^ Research Laboratory, Molecular Bases of Human Pathology, LR19ES13 Faculty of Medicine University of Sfax Sfax Tunisia; ^3^ High Institute of Sport and Physical Education of Sfax University of Sfax Sfax Tunisia; ^4^ Interdisciplinary Laboratory in Neurosciences, Physiology and Psychology: Physical Activity, Health and Learning (LINP2), UFR STAPS Paris Nanterre University Nanterre France; ^5^ Cardiovascular Surgery Unit Habib Bourguiba University Hospital, Faculty of Medicine, University of Sfax Sfax Tunisia; ^6^ Cardiology Unit Hedi Chaker Hospital University, Faculty of Medicine University of Sfax Sfax Tunisia

**Keywords:** acute myocardial infarction, cardiac autonomic function, physical activity, sleep

## Abstract

**Background:**

Monitoring lifestyle habits and physiological metrics are essential for improving cardiovascular outcomes and supporting recovery after acute cardiac events. Sleep is acknowledged as a core component of cardiovascular health and a predictive tool of adverse outcomes following acute myocardial infarction (AMI).

**Objective:**

The present study aimed to assess sleep metrics and explore the links between sleep patterns, heart rate variability (HRV) parameters, and traditional cardiovascular risk factors in patients with AMI.

**Methods:**

Sixty male patients with AMI (56.77 ± 8.24 years) participated in this study. Cardiac autonomic function was assessed with short‐term HRV analysis during the second week post‐AMI. Physical activity level was assessed using accelerometers. Sleep quality and quantity were evaluated objectively using a wrist‐worn accelerometer and subjectively by the Pittsburgh Sleep Quality Index. Chronotype was evaluated with the Horne and Otsberg questionnaire.

**Results:**

Twenty post‐AMI patients (33.3%) tended to experience poor sleep quality, with a sleep efficiency inferior to 85%. Thirty patients (50%) experienced short sleep duration, 16 (26.7%) had a healthy sleep duration (7−8 h), and 14 (23.3%) slept more than 8 h. Multiple regression analysis revealed that patients with healthy sleep quality and quantity exhibited higher HRV parameters, both in time and frequency domain values (*p* < 0.05). Low physical activity level was observed in patients with long sleep duration (*p* = 0.005) and evening chronotype (*p* = 0.022).

**Conclusion:**

Patients who spent more time performing moderate to vigorous physical activity tended to exhibit good sleep health and increased parasympathetic activity which are considered cardioprotective after AMI.

**Trial Registration:** PACTR202208834230748.

## Graphical abstract

During the second week after acute myocardial infarction treated With primary angioplasty, 60 patients were assessed for objective and subjective sleep quality, physical activity, chronotype, and heart rate variability. Poor sleep health was associated With lower physical activity levels and cardiac sympathetic overactivity, underscoring sleep′s prognostic relevance in post‐infarction care.

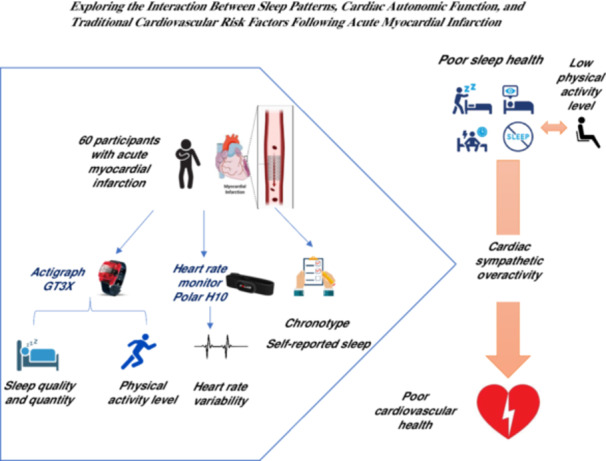

## 1 Introduction

According to the latest update of the American Heart Association in 2024, ischemic heart disease is the leading cause of cardiovascular disease (CVD) related deaths worldwide [1].

The etiology of acute myocardial infarction (AMI) is multifactorial, involving disruption in various aspects of cardiovascular health that gathers health behaviors (i.e., smoking, overweight, low physical activity level, and unhealthy diet) and health factors (i.e., high blood pressure, diabetes mellitus, and dyslipidemia) [2]. In a recent update by the American Heart Association in 2022, sleep has been recognized as the eighth major factor of cardiovascular health [3]. Cardiovascular processes, including heart rate, blood pressure, platelet activity, vagal modulation, cardiomyocyte function, and endothelial cells display circadian rhythm regulated by sleep/wake and fasting/feeding cycles under the control of circadian clock genes [4].

Patients with coronary artery disease often experience sleep disturbances, potentially due to circadian misalignment and impaired melatonin secretion regulated by the suprachiasmatic nucleus [5]. A reduction in melatonin secretion disrupts the circadian regulation of physiological processes, leading to a desynchronization of the sleep‐wake cycle and poor sleep quality and quantity [6]. Numerous epidemiological studies have demonstrated that poor sleep health is *a major* non‐traditional cardiovascular risk factor [7]. In this context, a recent 2024 review showed that poor sleep quality and insufficient or excessive sleep duration were associated with increased systemic inflammation and reduced cardiac vagal modulation [7]. In this line, sleep disorders are specifically associated with a poor prognosis following AMI and an increased risk of recurrent cardiac events and stroke [8]. Notably, a study of 1152 participants found that sleep disturbances remained a significant predictor of all‐cause mortality even 1 year later following AMI [9].

For assessing cardiac autonomic function, heart rate variability (HRV), the beat‐to‐beat difference in heart rate, is widely used as a noninvasive tool. High HRV reflects an increase in cardiac parasympathetic activity [10]. In contrast, low HRV reflects high cardiac sympathetic regulation [11]. Low HRV is associated with a higher risk of the onset of cardiovascular events. In contrast, an increase of 1% in the standard deviation of the normalized RR interval, a measure reflecting cardiac parasympathetic regulation, can reduce cardiovascular events by 1% [12]. Following AMI, HRV can predict cardiac mortality, arrhythmia, and sudden cardiac death [13]. It is important to maintain the electrical stability of the ventricular myocardium by sympathovagal balance to prevent ventricular arrhythmias [14].

Despite the growing recognition of sleep as a cardiovascular risk factor, the relationship between sleep characteristics and HRV following AMI remains poorly understood. Notably, most existing studies rely solely on subjective sleep measures (e.g., Pittsburgh Sleep Quality Index (PSQI), lacking objective sleep assessments.

The present study sought to bridge the gap in the existing literature by focusing on patients with AMI treated with primary percutaneous coronary intervention (PCI). The main objectives of the present study were to evaluate different sleep metrics (i.e., sleep duration, sleep quality, and chronotype) using objective and subjective measures, as well as to investigate the potential link between short‐term HRV and sleep patterns. In addition, the present study aimed to explore whether sleep disorders are associated with traditional cardiovascular risk factors.

## 2 Methods

### 2.1 Study Design

This observational study was conducted in the Department of Cardiology at Hedi Chaker University Hospital in Sfax (Tunisia), from December 2022 to September 2023. Before enrollment in the present study, all participants were informed about the study protocol and provided written informed consent. Ethical approval for the present study was obtained from the local Ethic and Investigation Committee (CPP SUD N° 0331/2021) in Tunisia. The study protocol was registered in the Pan African Clinical Trial Registry under the trial ID: PACTR202208834230748. The present study was conducted according to the Declaration of Helsinki.

### 2.2 Participants

This study included patients with a confirmed diagnosis of acute ST Elevation Myocardial Infarction (STEMI) and non‐STEMI treated with primary PCI. The diagnosis of STEMI or NSTEMI was established based on clinical presentation, electrocardiographic changes, and elevated cardiac biomarkers, in accordance with current international guidelines [15].

Patients were excluded if they had a history of atrial fibrillation, significant arrhythmias, or pacemaker implantation.

### 2.3 Sleep Quality and Quantity

Sleep quality and quantity were assessed objectively using the ActiGraph GT3X (ActiGraph Inc., Pensacola, FL, USA). The use of wrist‐worn accelerometers is validated to assess sleep metrics in comparison to polysomnography [16]. The ActiGraph GT3X serves to detect sleep and wakefulness periods by wrist movements during the night [17]. All patients wore the accelerometer during the second week following primary PCI. All accelerometers were initialized to a sampling rate of 30 Hz. The start time was set to mid‐day on the first day and the stop time was set to mid‐day on the seventh day. Actilife software (version 6.8.1, ActiGraph LLC, Pensacola, FL, USA) was used for the data exportation. Data were extracted in 60‐s epochs to optimize the signal‐to‐noise ratio [18]. The Cole‐Kripke algorithm was used to measure sleep efficiency, sleep duration, wake‐after‐sleep onset, and total time in bed [19].

Every night, the sleep period detected with the accelerometer′s algorithm was compared to the sleep interval mentioned in the patient's sleep diary. If there was more than a 30‐min difference in sleep intervals, sleep data (the time in bed and time out of bed) were modified in ActiLife according to the sleep diary. Daytime naps were not included in the sleep analysis.

To ensure compliance, participants received verbal instructions on the wrist‐worn accelerometer use. Furthermore, device wear time was visually verified using ActiLife's algorithm. To be considered a valid day of wear, participants were required to wear the accelerometer for a minimum of 10 h [20]. Participants with less than 4 days of valid data were excluded from the study.

To complement the objective assessment, the PSQI questionnaire was administered as a subjective tool to assess self‐reported sleep patterns. The PSQI index is the sum of seven components, each equally scored on a 0–3 scale: subjective sleep quality, sleep duration, sleep latency, sleep efficiency, use of sleep medication, sleep disturbances, and daytime dysfunction. Patients with a PSQI index above five were considered to have poor sleep quality [21].

### 2.4 Chronotype

Chronotype refers to the phenotypic expression of the circadian rhythm. It reflects the individual's preference for the time of day when they prefer to sleep and engage in daily activities. Chronotype was assessed via the Horne and Ostberg questionnaire, a valid tool in Arabic, composed of 19 items for measuring the morningness‐eveningness dimension reflecting the peak time of the circadian phase [22].

### 2.5 HRV

Short‐term HRV was measured in the supine position for 5 min using a Polar H10 heart rate monitor (Polar Electro Oy, Kempele, Finland) during the second week after hospital discharge. All HRV recordings were performed between 12:00 and 2:00 p.m. to minimize the influence of circadian variations on cardiac autonomic activity. Patients were instructed to abstain from consuming caffeine and alcohol for at least 12 h before the assessment.

According to the Task Force of the European Society of Cardiology and the North American Society of Pacing and Electrophysiology, the last 256 s of the 5‐min heart rate recordings were used for HRV analysis, including time‐domain analysis and frequency‐domain analysis [11]. Time‐domain analysis includes the standard deviation of RR intervals (SDNN) and the root mean square of successive differences between normal heartbeats (RMSSD). Regarding frequency‐domain analysis, a Fast Fourier Transform was used to quantify the power spectral density of low frequency (LF) and high frequency (HF) expressed in milliseconds squared. Data were imported into Kubios HRV Scientific 4.0 to detect noise and ensure beat correction using the “automatic method” [23].

### 2.6 Physical Activity

A wrist‐worn three‐axis accelerometer (ActiGraph GT3X, ActiGraph Inc, Pensacola, FL, USA) was used to measure physical activity levels. Data were downloaded and extracted using Actilife software (version 6.8.1; ActiGraph LLC, Pensacola, FL, USA).

Vector magnitude counts per minute (CPM) were utilized to calculate the time spent in moderate to vigorous intensity physical activity (MVPA), in light physical activity (LPA), as well as measuring sedentary time. In MVPA, the CPM is superior to 2690. In LPA, the CPM ranges between 200 and 2689. In sedentary time, the CPM is inferior to 200. These cutoff values have been validated in previous studies [24, 25].

## 3 Statistical Analyses

The Gaussian distribution was assessed by the Shapiro−Wilk test.

The null hypothesis of an absence of difference between groups in categorical variables regarding clinical characteristics was tested using the Chi 2 test.

The null hypothesis of an absence of difference between groups was tested through a one‐way ANOVA when data were distributed in a Gaussian way, or with a Kruskal−Wallis test when they did not fulfill this condition. When the null hypothesis was rejected, the Bonferroni post hoc test was used for pairwise comparisons. For one‐way ANOVA or Kruskal−Wallis analysis, the effect size was calculated using the partial eta square to estimate the significance of the findings. Small, moderate, and large effect sizes were represented by ηp2 values of 0.01, 0.06, and 0.13, respectively.

Multiple regression was used to explore whether clinical characteristics (i.e., age, body mass index, dyslipidemia, diabetes mellitus, glycated hemoglobin, dyspnea, family history of coronary artery disease, smoking, and New York Heart Association [NYHA] class) could impact HRV parameters and sleep metrics. It also explored the association between sleep patterns and HRV parameters. An *α* risk of 5% was retained for all analyses.

Statistical analyses were performed using SPSS (Statistical Package for the Social Sciences) version 26 software (SPSS Inc., Chicago, IL, USA).

## 4 Results

### 4.1 Participants

Sixty male post‐AMI patients with a mean age of 56.77 ± 8.24 years were included in this study. All demographic and clinical characteristics are shown in Table 1.

**Table 1:** Participants' demographic and clinical characteristics.

Demographic/clinical measures	Mean ± SD or *n* (%)
New York Heart Association Class 1	31 (51.7%)
New York Heart Association Class 2	24 (40%)
New York Heart Association Class 3	5 (8.3%)
Body mass index (kg/m^2^)	27.1 ± 3.6
Smoking (pack years)	32.6 ± 30.9
High blood pressure	36 (60%)
Dyslipidemia	45 (75%)
Dyspnea	30 (50%)
Diabetes mellitus	40 (66.7%)
Glycated hemoglobin (%)	7.25 ± 1.9
Family history of coronary artery disease	32 (53.3%)
Left ventricular ejection fraction (%)	53.6 ± 8.9
RR interval (ms)	930.9 ± 152.7
Standard deviation of RR intervals (ms)	20.3 ± 13.8
Root mean square of successive differences between normal heartbeats (ms)	22.2 ± 18.8
Low frequency (LF) (ms²)	234.2 ± 330.7
High frequency (HF) (ms²)	244.4 ± 496.1
LF/HF ratio	2.1 ± 2.1
Moderate physical activity (min/week)	184.7 ± 85.9
Moderate physical activity (%)	14.7 ± 6.8
Light physical activity (min/week)	1069.3 ± 84.2
Light physical activity (%)	85.31 ± 6.78
Total sedentary time (min/week)	724.2 ± 104.53
Ricci et Gagnon score	12.13 ± 2.49

*Note:* Data are presented as mean ± standard deviation (SD) or as number (*n*).

### 4.2 Sleep Quality and Sleep Duration

Patients with AMI tended to experience poor sleep quality with a mean PSQI of 10.2 (Table 2). 20 patients (33.3%) had poor sleep quality with a sleep efficiency < 85%. Thirty patients (50%) had short sleep duration (< 7 h), 14 patients (23.3%) experienced long sleep duration (> 8 h), and 16 (26.7%) had healthy sleep duration (7−8 h).

**Table 2:** Sleep characteristics.

Sleep parameters	Mean ± standard deviation
Total time in bed (min)	463,6 ± 93.4
Sleep duration (min)	412.6 ± 80.3
Sleep efficiency (%)	87.6 ± 5.7
Wake after sleep onset (min)	61.1 ± 39.6
Pittsburgh sleep quality Index score	10.2 ± 3.3

### 4.3 Effect of Clinical Factors on Sleep Patterns and HRV Parameters

Multiple regression analysis showed that none of the clinical characteristics affected sleep patterns and HRV parameters, as presented in Supporting Informatin S1: Table 1.

### 4.4 Time Domain HRV Parameters and Sleep Metrics

Multiple regression analysis showed that SDNN and RMSDD were influenced by sleep efficiency and sleep duration (Table 3).

**Table 3:** Multiple regression analysis between sleep patterns and time‐domain analysis parameters.

	SDNN				RMSSD			
Model	Adjusted *R* ^2^ = 0.217, *F* = 4.269, *p* = 0.002				Adjusted *R* ^2^ = 0.241, *F* = 3.436, *p* = 0.009			
	*B*	SE	*β*	*t*	*B*	SE	*β*	*t*
Sleep efficiency	1.448	0.432	0.599	3.350*	1.605	0.604	0.489	2.265*
Time in bed	0.171	0.05	1.196	−3.401*	0.232	0.07	1.198	3.311*
Total sleep time	−0.166	0.061	−0.964	−2.706*	−0,220	0.869	−0.942	−2.569
Wake‐up after sleep onset	−0.029	0.052	−0.082	−0.533	−0.048	0.072	−0.1	−0.656
Chronotype	0.135	0.126	0.129	1.076	0.204	0.176	0.143	1.162

Abbreviations: *B* = unstandardized regression coefficient; *β* = standardized coefficient; *p* = level of statistical significance; PSQI = Pittsburgh Sleep Quality Index; *R* ^2^ = coefficient of determination; RMSSD = root mean square of successive differences between normal heartbeats; SDNN = standard deviation of RR intervals; SE = standard error; *t* = *t* statistic.

* Significant difference at *p* < 0.05.

### 4.5 Frequency Domain HRV Parameters and Sleep Metrics

Multiple regression analysis showed that HF was affected by sleep efficiency and total sleep duration (Table 4). However, LF and LF/HF ratio were not associated with sleep patterns.

**Table 4:** Multiple regression analysis between sleep patterns and frequency‐domain analysis parameters.

	High frequency			
	Adjusted *R* ^2^ = 0.160, *F* = 3.549, *p* = 0.02			
	*B*	SE	*β*	*t*
Sleep efficiency	31.850	15.020	0.366	2.12*
Total time in bed	4.587	1.886	0.894	0.243
Total sleep time	−3.873	2.327	−0.627	−1.664*

Abbreviations: *B* = unstandardized regression coefficient; *β* = standardized coefficient; *p* = level of statistical significance; *R* ^2^ = coefficient of determination; SE = standard error; *t* = *t* statistic.

* Significant difference at *p* < 0.05.

Patients with low sleep efficiency and short sleep duration had lower values of RMSSD, SDNN, and HF.

### 4.6 Physical Activity Level

Twenty‐two patients (36.6%) did not meet the recommended threshold of 150 min per week of MVPA.

### 4.7 Sleep Duration and Cardiovascular Risk Factors

**Figure 1:**
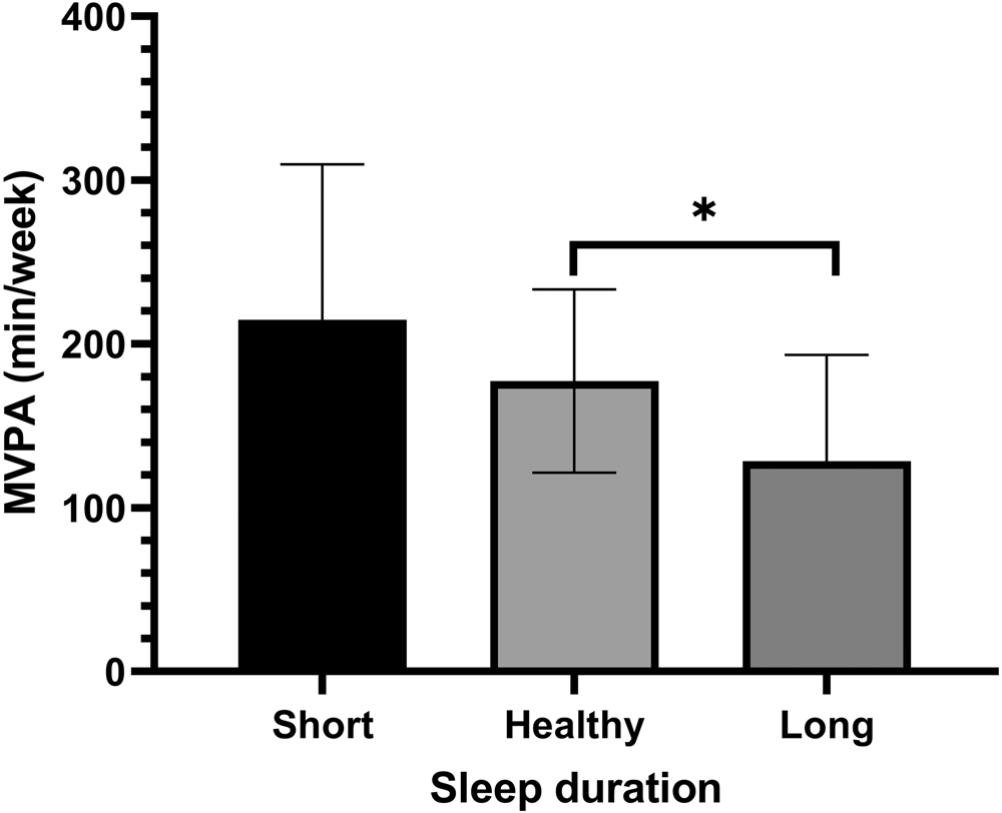
Moderate to vigorous physical activity by sleep duration. *Significant difference at *p* < 0.01; MVPA, moderate to vigorous physical activity.

A difference in MVPA was found between sleep duration groups (*H* = 9.998, *p* = 0.007, *η* ^2^ = 0.14). The Bonferroni post‐hoc test revealed that patients with long sleep duration spent less time in MVPA than those with healthy sleep duration (*p* = 0.005) (Figure 1).

### 4.8 Chronotype and Cardiovascular Risk Factors

**Figure 2:**
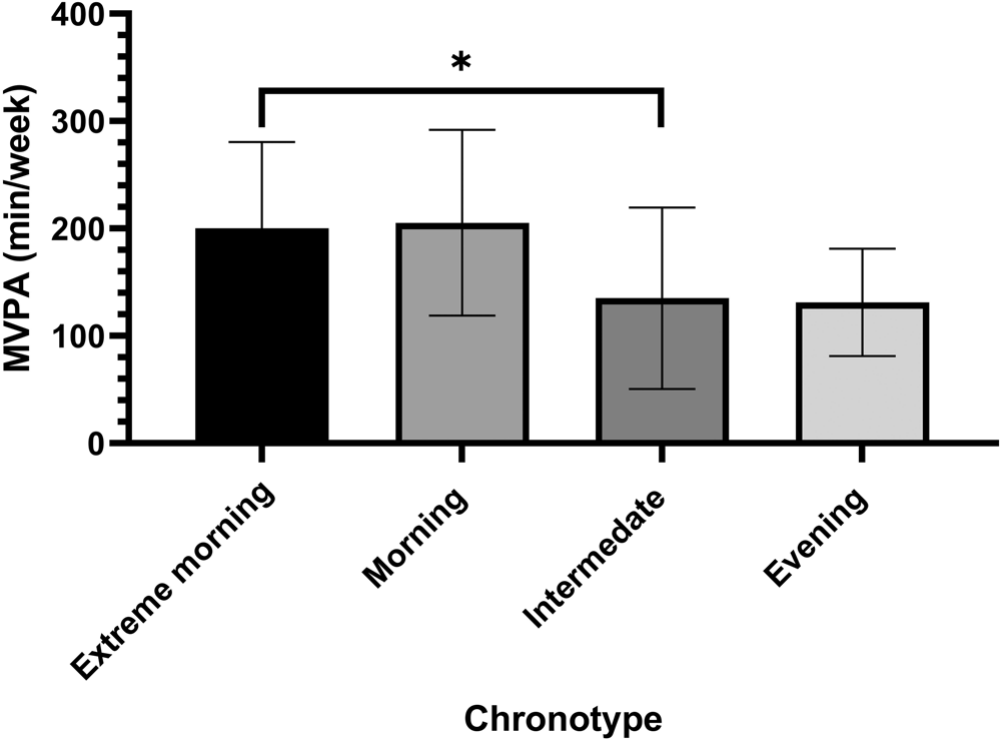
Moderate to vigorous physical activity by chronotype. *Significant difference at *p* < 0.05; MVPA, moderate to vigorous physical activity.

A difference in MVPA was found between chronotype groups (*F*_(3, 56)_ = 2.808, *p* < 0.05, η2 = 0.13). Patients with the morning chronotype were more active than those with the intermediate chronotype (*p* = 0.022) (Figure 2).

## 5 Discussion

To the best of our knowledge, this is the first study that investigated the association between sleep metrics, cardiac autonomic function, and traditional cardiovascular risk factors, including physical activity level in patients with AMI. Our findings confirmed that poor sleep quality and quantity were associated with an imbalance between cardiac parasympathetic and sympathetic activity. Furthermore, low physical activity level was observed in patients with long sleep duration and intermediate chronotype.

### 5.1 Sleep Duration

The findings of this observational study revealed that 50% of patients with AMI slept less than 7 h, while 23.3% slept more than 8 h. Our results were similar with the findings of the SOLID‐TIMI 52 trial, revealing that patients with AMI reported short sleep duration [26].

The association between nighttime sleep and CVD follows a *U*‐shaped pattern. A sleep duration inferior to 7 h increases the risk of developing AMI by 15% [27]. For each additional hour of sleep, the risk of AMI decreased by 20%. In this line, short sleep duration is considered a potential CV risk factor and is associated with an increased mortality rate [28]. The risk of recurrent AMI and death in the year following AMI could increase by 50% in patients reporting short sleep duration [29]. On the other hand, sleep duration exceeding 9 h is associated with a 30% increased risk of incident AMI [30]. A large meta‐regression analysis of 40 prospective cohort studies, showed that both short and long sleep duration are associated with an increased mortality rate [31]. Likewise, a most recent prospective study among healthy subjects found that long sleep duration is associated with an increased risk of CV events, particularly when it is accompanied by at least one cardiovascular risk factor, such as overweight, hyperglycemia, or dyslipidemia [32]. In this line, the Morgen research [33] and the UK Biobank cohort research found that a recommended sleep duration of 7−8 h can protect against cardiovascular events and decrease the mortality rate [34].

### 5.2 Sleep Quality After AMI

In this study, we found that post‐AMI patients reported poor sleep quality with a PSQI score of 10.1 ± 3.4. Our results were comparable with a prospective observational study revealing that patients with AMI had sleep disorders with a mean PSQI score of 6.7 ± 4.4 [35]. In this line, a recent systematic review assessing sleep quality using PSQI score in patients following AMI found that poor sleep quality was observed immediately after acute coronary syndrome [36].

In our study, an accelerometer was used to measure sleep quality objectively. We found that mean sleep efficiency was 87.1% and 20 patients (33.3%) had poor sleep quality with a sleep efficiency < 85%. In this context, a study performed on hospitalized patients with AMI revealed that sleep efficiency was 77.3% [37]. In this line, a study found that patients following acute coronary syndrome presented poor sleep quality with a sleep efficiency of 84.6% in the cardiac care unit and 83.1% in the ward assessed on the fourth day following acute coronary syndrome [38].

### 5.3 Chronotype in Post‐AMI Patients

Another sleep pattern investigated in this study was chronotype. It is important to assess chronotype in this population because it reflects the desynchronization between the internal clock and external factors caused by changes in meal timing and sleep patterns. In our study, 73.3% of the patients with AMI had a morning chronotype. Our results were in contrast with the literature demonstrating that individuals with the evening chronotype, such as shift workers, are more susceptible to developing CVD than those with the morning chronotype [39]. In addition, the evening chronotype was associated with an increased 10‐year risk of the first onset of a CV event [40]. Interestingly, it has been previously demonstrated that morning chronotype was associated with low CVD risk factors in the UK biobank cohort study [41]. Therefore, we suggest that morning chronotype can′t be protective against CVD if it is associated with sleep disorders.

### 5.4 Sleep Patterns and HRV

The present study aimed to understand how different sleep metrics (i.e., sleep quantity and quality, and chronotype), could impact the balance between cardiac sympathetic and parasympathetic activity.

It has been proven that HRV is a predictor of mortality following AMI [42]. Low SDNN values reflect the poor prognostic following AMI. It has been considered a strong predictor of major adverse cardiac events after AMI [43]. However, high values of SDNN and RMSSD after primary PCI reflect the increase of parasympathetic activity and the restoration of the balanced autonomic function [44].

LF is related to baroreflex activity [45]. The HF component is widely used as a specific marker of parasympathetic activity and reflects the vagal “tone.” Higher HF power indicates stronger parasympathetic influence and better autonomic balance [46]. The ratio LF/HF reflects sympathovagal balance [11]. Elevated LF/HF ratio suggests sympathetic overactivity and/or reduced parasympathetic activity. Imbalance in autonomic regulation has been associated with increased cardiovascular risk and adverse cardiac events [47].

Our current findings showed low sleep efficiency and short sleep duration were associated with low parasympathetic activity marked by low values in RMSSD, SDNN, and HF. Our results were comparable with a study revealing that PSQI score was correlated with lower SDNN [48]. In this line, it was demonstrated that patients with obstructive sleep apnea had low HF and SDNN values [49].

The association between sleep metrics and cardiac autonomic function can be explained by the existence of a circadian rhythm of HRV under the control of the hypothalamic‐pituitary‐adrenal axis [50]. HF exhibited circadian variation with a peak between 7:00 and 8:00 a.m. and a second peak that coincides with cardiovascular events around 10:00 p.m. [51]. In healthy subjects, vagal tone peak plays a crucial role in cardioprotection from the rapid rise of catecholamine, responsible for blood clotting, vasoconstriction, and myocardial oxygen demand, leading to adverse cardiovascular events [52]. In this line, some studies highlighted that diurnal variation in sympathetic activity, related to catecholamine circadian fluctuation, plays a crucial role in driving the circadian rhythm of HRV, independent of vagal tone rhythmicity [53]. Thus, sleep disturbances contribute to a significant hormonal imbalance, with morning cortisol levels falling by 30% and afternoon levels rising by approximately 40%. This stress reaction results in elevated blood pressure, elevated heart rate, and low HRV [54].

Additionally, circadian desynchronization can be caused by reduced levels of melatonin production and secretion, as demonstrated in patients with coronary artery disease [5]. Melatonin is the key circadian synchronization hormone responsible for healthy sleep [55]. In turn, it has a cardioprotective effect against ischemia‐reperfusion injury characterized by the pro‐inflammatory response and oxidative stress exacerbation [56]. Notably, numerous studies have verified that melatonin is a potent scavenger of reactive oxygen species and has a powerful capacity to activate antioxidant enzymes [57]. In this context, it was well demonstrated that oxidative stress was linked with the overactivity of the sympathetic system characterized by low HRV [58].

Furthermore, the link between sleep health and HRV may be explained by the fact that the dominance of either the sympathetic or parasympathetic nervous system characterizes each sleep phase [59]. Any awakening during the night, regardless of the sleep phase, induces an imbalance in the autonomic nervous system and neurohormones, leading to overactivity of the sympathetic system throughout the 24‐h day, which increases the risk of cardiovascular events [60]. Interestingly, the link between sleep and HRV is bidirectional. Reduced vagal tone and increased sympathetic activity impair sleep initiation and fragment sleep architecture [61].

### 5.5 Sleep and Cardiovascular Risk Factors

In this study, patients recovering from AMI displayed sedentary behavior with a Ricci et Gagnon score of 12.13. However, during the first week post‐AMI, patients were encouraged to meet the World Health Organization standard recommendations (≥ 150 min of MVPA throughout the week) [62].

In this study, patients accumulated an average of 184.7 ± 85.9 min of MVPA per week during the first week following AMI, with 33.6% of them not reaching the World Health Organization′s recommended threshold. Interestingly, there is a lack of studies assessing physical activity levels objectively during the acute phase after myocardial infarction. To date, one study reported an average of 130 min per week of physical activity in post‐AMI patients, assessed using the International Physical Activity Questionnaire, a subjective self‐report tool that can over or underestimate actual activity levels due to recall bias [63].

Findings showed that patients with healthy sleep durations and morning chronotypes were more likely to engage in MVPA and transition to an active lifestyle than those with long sleep durations and intermediate chronotypes.

In this line, a most recent umbrella review demonstrated that long sleep duration is associated with sedentary behavior and lower physical activity levels [64]. A most recent cohort study has demonstrated that more time spent in MVPA was associated with a lower risk of mortality and major adverse cardiovascular events through benefits on cardiovascular function [65]. Additionally, a most recent study demonstrated that meeting the recommended threshold of MVPA can mitigate the adverse effects of both short and long sleep duration [66].

Our results align with a recent systematic review of 23 studies, which revealed that individuals with an evening chronotype tend to engage in less physical activity and spend more time being sedentary than those with a morning chronotype [67]. Awareness of the link between evening chronotype and the 10‐year risk of first‐onset CVD mediated by low physical activity levels [68] could motivate patients following AMI to change their lifestyle behavior by practicing physical activity and avoiding the delay of sleep time. Additionally, practicing physical activity not only enhances the cardiovascular system and reduces the rate of re‐hospitalization following AMI, but also it is a potential zeitgeber contributing a beneficial effect on circadian clock resynchronization [69].

The strength of our study lies in exploring the association between sleep metrics, traditional cardiovascular risk factors, and cardiac autonomic function following AMI using an accelerometer, thereby eliminating the recall bias associated with self‐reported questionnaires. However, this study has several limitations, such as a reduced sample size, it was a single‐center study. In this study, chronotype was evaluated using a questionnaire. It can be better assessed by measuring dim light melatonin onset. Furthermore, specific details regarding specific environmental and social elements (e.g., shift work, social jetlag, and light exposure) that may be impacting chronotype should be provided. Furthermore, oxidative stress is a crucial factor in understanding the relationship between HRV and sleep disturbances. However, the association between oxidative stress and sleep patterns was not investigated in the present study. Moreover, measuring HRV during different sleep stages would be beneficial to provide a more comprehensive analysis of HRV and its association with sleep.

## 6 Conclusion

This study aimed to evaluate various sleep metrics and investigate the relationship between sleep patterns and HRV parameters. Additionally, it sought to determine whether traditional cardiovascular risk factors are associated with poor sleep health. Key findings revealed that post‐AMI patients commonly experience disruptions in both sleep quality and duration. Notably, better sleep quality and adequate sleep duration were strongly associated with higher HRV parameters, including HF, RMSSD, and SDNN, indicating enhanced parasympathetic activity. These findings underscore the potential role of sleep in cardiovascular health. Furthermore, patients with healthy sleep durations and a morning chronotype were more likely to engage in MVPA. Therefore, promoting lifestyle changes such as optimizing sleep timing, maintaining adequate sleep duration, and adhering to recommended MVPA levels can enhance parasympathetic activity and potentially improve outcomes in patients recovering from AMI.

## Author Contributions

**Mohamed Ali Hbaieb:** investigation, conceptualization, methodology, formal analysis, data curation, writing − original draft. **Laurent Bosquet:** supervision, writing − review and editing. Omar Hammouda: supervision, conceptualization. Raghda Hbaieb: writing − review and editing. **Ines Mezghani:** resources. **Salma Charfeddine:** supervision, conceptualization. Leila Abid: resources. **Tarak Driss:** writing − review and editing. **Benoit Dugué:** supervision, writing − review and editing. All authors read and approved the manuscript.

## Conflicts of Interest

The authors declare no conflicts of interest.

## Supporting information

supmat.

## Data Availability

The data that support the findings of this study are available from the corresponding author upon reasonable request.
