# Supplementary material for: Exploring the Interaction Between Sleep Patterns, Cardiac Autonomic Function, and Traditional Cardiovascular Risk Factors Following Acute Myocardial Infarction
Source: Clin Cardiol. 2025 Jul 24;48(7):e70183. doi: 10.1002/clc.70183 (PMC12287801; doi:10.1002/clc.70183)
Supplement: Supplementary file 1 — supmat. [file CLC-48-e70183-s001.docx]

Supplementary Table 1. Multiple regression analysis of sleep patterns and heart rate variability parameters.

|  | **F** | **p** | **Adjusted R2: proportion of variance** |
| --- | --- | --- | --- |
| **SDNN** | 1.337 | 0.242 | 0.049 |
| **RMSSD** | 1.348 | 0.237 | 0.05 |
| **LF** | 0.725 | 0.684 | −0.044 |
| **HF** | 1.437 | 0.198 | 0.062 |
| **LF/HF** | 0.596 | 0.794 | −0.066 |
| **PSQI** | 0.846 | 0.578 | −0.024 |
| **Sleep efficiency** | 0.541 | 0.815 | −0.07 |
| **Sleep duration** | 0.649 | 0.75 | −0.05 |
| **Total time in bed** | 0.455 | 0.898 | −0.091 |
| **WASO** | 0.466 | 0.903 | −0.092 |

SDNN: Standard deviation of RR intervals; RMSSD: root mean square of successive differences between normal heartbeats; HF: high frequency; LF: low frequency; PSQI: Pittsburgh Sleep Quality Index; WASO: wake after sleep onset.
